# Aspirin and risk for gastric cancer: a population-based case–control study in Sweden

**DOI:** 10.1054/bjoc.2001.1702

**Published:** 2001-04

**Authors:** K Akre, A M Ekström, L B Signorello, L-E Hansson, O Nyrén

**Affiliations:** Department of Medical Epidemiology, Karolinska Institutet, 171 77 Stockholm, Sweden; International Epidemiology Institute, 1550 Research Boulevard, Rockville, MD 20850, USA; Mora Hospital, S-792 37 Mora, Sweden

**Keywords:** gastric cancer, aspirin, NSAIDs, *Helicobacter pylori*

## Abstract

While aspirin and other non-steroid anti-inflammatory drugs (NSAIDs) are associated with gastric mucosal damage, they might reduce the risk for gastric cancer. In a population-based case–control study in 5 Swedish counties, we interviewed 567 incident cases of gastric cancer and 1165 controls about their use of pain relievers. The cases were uniformly classified to subsite (cardia/non-cardia) and histological type and information collected on other known risk factors for gastric cancer. *Helicobacter pylori* serology was tested in a subset of 542 individuals. Users of aspirin had a moderately reduced risk of gastric cancer compared to never users; odds ratio (OR) adjusted for age, gender and socioeconomic status was 0.7 (95% CI = 0.6–1.0). Gastric cancer risk fell with increasing frequency of aspirin use (*P* for trend = 0.02). The risk reduction was apparent for both cardia and non-cardia tumours but was uncertain for the diffuse histologic type. No clear association was observed between gastric cancer risk and non-aspirin NSAIDs or other studied pain relievers. Our finding lends support to the hypothesis that use of aspirin reduces the risk for gastric cancer. © 2001 Cancer Research Campaign http://www.bjcancer.com


British Journal of Cancer (2001) **84**(7), 965–968
© 2001 Cancer Research Campaign
doi: 10.1054/ bjoc.2001.1702, available online at http://www.idealibrary.com on IDEAL®

### Aspirin and risk for gastric cancer: a population-based case–control study in Sweden

**K Akre[1], AM Ekström[1], LB Signorello[2], L-E Hansson[3] and O Nyrén[1]**

[1]Department of Medical Epidemiology, Karolinska Institutet, 171 77 Stockholm, Sweden; [2]International Epidemiology Institute, 1550 Research Boulevard, Rockville, MD 20850, USA; [3]Mora Hospital, S-792 37 Mora, Sweden

**Summary** While aspirin and other non-steroid anti-inflammatory drugs (NSAIDs) are associated with gastric mucosal damage, they might reduce the risk for gastric cancer. In a population-based case–control study in 5 Swedish counties, we interviewed 567 incident cases of gastric cancer and 1165 controls about their use of pain relievers. The cases were uniformly classified to subsite (cardia/non-cardia) and histological type and information collected on other known risk factors for gastric cancer. *Helicobacter pylori* serology was tested in a subset of 542 individuals. Users of aspirin had a moderately reduced risk of gastric cancer compared to never users; odds ratio (OR) adjusted for age, gender and socioeconomic status was 0.7 (95% CI = 0.6–1.0). Gastric cancer risk fell with increasing frequency of aspirin use (*P* for trend = 0.02). The risk reduction was apparent for both cardia and non-cardia tumours but was uncertain for the diffuse histologic type. No clear association was observed between gastric cancer risk and non-aspirin NSAIDs or other studied pain relievers. Our finding lends support to the hypothesis that use of aspirin reduces the risk for gastric cancer. © 2001 Cancer Research Campaign http://www.bjcancer.com

**Keywords**: gastric cancer; aspirin; NSAIDs; *Helicobacter pylori*

While on the one hand causing damage to the gastrointestinal mucosa (Hawkey, 1996), aspirin and other nonsteroidal anti-inflammatory drugs (NSAIDs) also have some potentially anti-carcinogenic activity. These include repression of prostaglandin synthesis through inhibition of cyclooxygenase (COX) (Marnett, 1992; Lupulescu, 1996), induction of apoptosis (Shiff and Rigas, 1997) and inhibition of angiogenesis (Jones et al, 1999). Indeed, COX inhibitors have been reported to suppress growth of gastric cancer in a human gastric cancer cell line (MKN45) (Tsuji et al, 1996; Sawaoka et al, 1998c) and in gastric cancer xenografts transplanted into nude mice (Sawaoka et al, 1998b). Accumulating evidence of a clinically significant anticarcinogenic effect of aspirin has so far mainly pertained to colorectal cancer (Kune et al, 1988; Rosenberg et al, 1991; Giardiello et al, 1993; Thun et al, 1993; Schreinemachers and Everson, 1994), while data from the combined epidemiologic literature regarding gastric cancer and aspirin are less convincing (Isomaki et al, 1978; Gridley et al, 1993; Thun et al, 1993; Schreinemachers and Everson, 1994). Recently, 2 case–control studies indicated that the risk for stomach cancer is reduced in users of NSAIDs (Farrow et al, 1998; Zaridze et al, 1999).

Using data from a large population-based case–control study primarily concerned with diet and other life-style factors (Ekstrom et al, 2000), we aimed to further explore the possible protective effect of aspirin on gastric cancer. Available data enabled us to study this association for each tumour site (cardia/non-cardia) and histological subtype separately, while taking other risk factors, including *H. pylori*, into account.

*Received 20 September 2000*
*Revised 10 January 2001*
*Accepted 12 January 2001*

*Correspondence to:* K Akre

### METHODS

Study methods have been described in detail elsewhere (Ekstrom et al, 1999a). Briefly, the study base consisted of all native Swedes aged 40–79 years, living in 5 Swedish counties (population 1.3 million) at any time from February 1989 to January 1995. All gastric adenocarcinomas in the study base were eligible as cases.

### Study participants

A comprehensive scheme for case ascertainment, with contact persons at all hospitals, continuous surveillance at all pathology departments, and a final check at the regional cancer registers up to 30 months after the end of the study period, exceeded that of the Swedish Cancer Register (Ekstrom et al, 1999b). Of the 908 patients who met the eligibility criteria, 567 (62%) agreed to participate in a face-to-face interview. The reasons for non-participation were early death or terminal illness in 270 (29.7%), mental or physical illness other than gastric cancer in 40 (4.4%), patient refusal in 28 (3.1%), while 3 (0.3%) could not be located.

Tumour subsite and histologic type [according to Laurén (1965)] were determined using standardized reports from the clinicians, supplemented by data from the patient records. All histological slides were re-evaluated by one pathologist. Cancer of the gastric cardia ($n$ = 90) was defined as an adenocarcinoma with its centre located within one centimetre proximal and 2 centimetres distal to the gastro-oesophageal junction (Misumi et al, 1989). Tumours were classified according to their predominant histologic pattern as being of intestinal ($n$ = 337), diffuse ($n$ = 184), or mixed type ($n$ = 37). We could not determine the exact site of tumour origin or the histologic type in 0.9% and 1.6%, respectively.

The control subjects were randomly selected from a continuously updated population registry covering the entire study base. We frequency-matched the controls to mimic the age and gender distribution of cases. Of the 1534 contacted control subjects, 1165 were interviewed (76.0%). The reasons for non-participation were refusal in 245 (16.0%), mental or physical illness in 90 (5.9%), while 34 (2.2%) could not be located for interview.

### Exposure data

We obtained information through face-to-face interviews conducted by specially trained professional interviewers employed by Statistics Sweden. Concealment of case/control status was impossible, but the interviewers were instructed to treat cases and controls in a strictly uniform way. The interviewers were, furthermore, unaware of the study hypothesis. Questions were asked about use of pain relievers earlier than 2 years before the interview and the participants were prompted to give specific brand names. The subjects reported the frequency of pain reliever use per month and, when taking them, the number of tablets used per day. We defined ever users of aspirin as those who reported ever use of pain relievers containing salicylic acid with a frequency equal to or exceeding one tablet per month. Ever use of non-aspirin and other pain relievers was defined correspondingly. To obtain a measure of monthly dose, we multiplied (tablets per day) × (number of days using pain relievers per month). The distribution was skewed towards low intake, and to attain categories of reasonable size, while at the same time being able to examine a group that was significantly exposed, the monthly dose variable was transformed into categories of <1, 1–29, and ≥ 30. The median value in each category was 0.5, 2, and 60 tablets per month, respectively, and the highest category represented the 90th percentile of exposure.

During the last years of our study, we collected serum samples for the study subjects (297 cases and 245 controls). Serum IgG antibodies to *H. pylori* were determined using an enzyme-linked immunosorbent assay (HM Cap®) with a reported sensitivity and specificity of 94–98% (Graham and Evans 1994) and 92–97% (Marchildon et al, 1996), respectively.

For the multivariate analyses, we considered as covariates a weighted life-time average of socio-economic status divided into 5 categories (Hansson et al, 1994), smoking habits (non-smoker, cigarette smoker for <30 years, cigarette smoker for ≥30 years), *H. pylori* serostatus (positive vs. negative), intake of fruit and vegetables (above or below the median among controls) and upper gastrointestinal symptom history (negative vs. positive defined as ever-use of $H_2$-receptor antagonists or physician-diagnosed peptic or gastric ulcers).

### Statistical methods

We computed odds ratios (OR) and 95% confidence intervals (CI) by using unconditional logistic regression. Model parameters were estimated by the maximum likelihood method (Hosmer and Lemeshow, 1989). All main effect models included the frequency matching factors age and gender. We tested for linear trend by constructing ordinal variables through assigning consecutive integers to consecutive levels of the categorized variables (Wald's test). Two-sided $P$ values were used in all analyses.

### RESULTS

We excluded 37 cases and 25 controls because they reported a previous subtotal gastrectomy. Relevant data on use of pain relievers was missing or incomplete for 50 cases and 85 controls. For 71 cases and 137 controls that reported multiple drug use, we did not obtain data on drug-specific doses. Therefore, some analyses (including those evaluating possible dose effects) were restricted to individuals for which this information was available.

Selected characteristics of case and control subjects are presented in Table 1. The male/female ratio was 2:1. Mean age for cases and controls was 67.8 and 66.9 years, respectively. The corresponding data for the group tested for *H. pylori* status was 68.2 and 67.1 years.

Table 2 presents unconditional logistic regression-derived odds ratios for the association between use of aspirin and gastric cancer risk. We observed a moderately strong inverse association between aspirin use and gastric cancer, indicating a 20–30% lower risk among aspirin users. The risk deficit was less convincing for non-cardia cancer of the diffuse type. Gastric cancer risk fell with increasing frequency of aspirin use ($P$ for trend = 0.02), and those who used at least 30 tablets per month or more had a 40% (ns) lower risk than those who used less than 1 tablet per month (Table 3). Use of non-aspirin NSAIDs was not associated with a lower risk of gastric cancer (OR = 1.1, 95% CI = 0.6–1.4), although the small number of exposed individuals (27 cases and 54 controls) made the estimate unstable. We further explored use of other pain relievers (paracetamol and dextropropoxifen) and found no association with gastric cancer risk (data not shown).

Adjusting for socioeconomic status, cigarette smoking, upper gastrointestinal symptoms and intake of fruit and vegetables did not change our results materially, nor did controlling for *H. pylori* infection within the group for which data on *H. pylori* status was

**Table 1** Characteristics of 567 cases of gastric cancer and 1165 control subjects generated in a population of 5 countries in Sweden

| | Cases (%) | Controls (%) |
|---|---|---|
| Age | | |
| 40–59 | 103 (19) | 201 (18) |
| 60–69 | 155 (29) | 363 (32) |
| 70–79 | 272 (51) | 576 (50) |
| Gender | | |
| Female | 185 (35) | 381 (33) |
| Male | 345 (65) | 759 (67) |
| Cigarette smoking[b] | | |
| Non-smokers | 255 (48) | 592 (52) |
| <30 years[a] | 108 (20) | 275 (24) |
| ≥30 years | 167 (32) | 269 (24) |
| Socioeconomic status[b] | | |
| Unskilled manual workers | 259 (49) | 441 (39) |
| Skilled manual workers | 124 (23) | 260 (23) |
| Non-manual workers | 90 (17) | 295 (26) |
| Self-employed ≥19 years | 23 (4) | 54 (5) |
| Farmers ≥19 years | 33 (6) | 89 (8) |
| *Helicobacter pylori* infection[c] | | |
| No | 80 (28) | 107 (45) |
| Yes | 201 (72) | 132 (55) |

[a] Median duration of cigarette smoking among controls who were smokers.
[b] Non-additivity due to missing data on smoking ($n$ = 4) and socioeconomic status ($n$ = 2). [c] *H. pylori* serology was assessed for a subset of the population ($n$ = 520).

© 2001 Cancer Research Campaign

**Table 2** Odds ratios (ORs)a and 95% confidence intervals (CIs) for use of aspirin in relation to gastric cancer, by site and histologic subtype

| | | All gastric cancer | | Cardia | | Non-Cardia | | | | | |
|---|---|---|---|---|---|---|---|---|---|---|---|
| | | | | | | Non-Cardia | | Intestinal | | Diffuse | |
| | Controls | Cases | OR (95% CI) | Cases | OR (95% CI) | Cases | OR (95% CI) | Cases | OR (95% CI) | Cases | OR (95% CI) |
| Use of asprin[b] | | | | | | | | | | | |
| No | 624 | 306 | (Ref.) | 53 | (Ref.) | 249 | (Ref.) | 141 | (Ref.) | 85 | (Ref.) |
| Yes | 294 | 103 | 0.7 (0.6–1.0) | 15 | 0.6 (0.3–1.1) | 88 | 0.8 (0.6–1.0) | 48 | 0.8 (0.5–1.1) | 32 | 0.9 (0.6–1.3) |
| Use of aspirin[b,c] | | | | | | | | | | | |
| No | 631 | 310 | (Ref.) | 53 | (Ref.) | 253 | (Ref.) | 143 | (Ref.) | 86 | (Ref.) |
| Yes | 424 | 170 | 0.8 (0.7–1.1) | 25 | 0.7 (0.4–1.2) | 145 | 0.9 (0.7–1.1) | 74 | 0.8 (0.6–1.1) | 62 | 1.1 (0.8–1.5) |

[a]Adjusted for age, gender and socioeconomic status. [b]Defined as equal to or exceeding one tablet per month. [c]Including subjects with missing specific dosage information ($n$ = 208). All information on use of pain relievers was missing for 135 individuals.

**Table 3** Odds ratios (ORs)[a] and 95% confidence intervals (CIs) for use of aspirin in relation to gastric cancer

| | Controls ($n$ = 409)[d] | Cases ($n$ = 918)[d] | OR[a] |
|---|---|---|---|
| Number of tablets per month[b] | | | |
| <1 | 306 | 624 | 1.0 (ref.) |
| 1–29 | 89 | 247 | 0.8 (0.6–1.0) |
| ≥30[c] | 14 | 47 | 0.6 (0.3–1.1) |
| $P$ (trend) | | | 0.02 |

[a]Adjusted for age, gender and socioeconomic status. [b]Daily dose multiplied by frequency of use per month. [c]90th percentile of the exposed subjects. [d]Missing information on use of pain relievers ($n$ = 135) and drug-specific doses ($n$ = 208).

**Table 4** Distribution of cases with gastric cancer and controls by *Helicobacter pylori* by status and aspirin use

| | Aspirin use[b] | | |
|---|---|---|---|
| | Group | No | Yes |
| *Helicobacter pylori* positive | cases | 124 | 65 |
| | controls | 68 | 53 |
| | OR[a] (95% CI) | 1.0 (ref.) | 0.6 (0.4–1.0) |
| *Helicobacter pylori* negative | cases | 45 | 22 |
| | controls | 58 | 38 |
| | OR[a] (95% CI) | 1.0 (ref.) | 0.8 (0.4–1.5) |

[a]Adjusted for age, gender and socioeconomic status. [b]Defined as equal to or exceeding one tablet per month.

available. Use of aspirin was similar for subjects with and without reported upper gastrointestinal tract symptoms (61% and 60% for those with and without physician-diagnosed peptic or duodenal ulcers, respectively, and 58% and 60% for users and non-users of $H_2$-blockers, respectively).

Among the subjects who were tested for *H. pylori* serology, we performed a stratified analysis according to serostatus. Although hampered by small numbers, this analysis revealed a non-significant tendency for effect modification. Among the *H. pylori*-positive subjects the risk was reduced by 40%, whereas the risk reduction among the *H. pylori*-negative subjects was only 20% and statistically non-significant. The difference in risk between *H. pylori* strata was, however, not statistically significant, nor was the interaction term in our full logistic regression model.

### DISCUSSION

In this large population-based case-control study, we found a moderately strong inverse and apparently dose-dependent association, although only of borderline statistical significance, between use of aspirin and gastric cancer risk. This inverse association was apparent for both cardia and non-cardia cancer, but uncertain for non-cardia cancer of the diffuse type.

A few cohort studies have investigated the relation between use of NSAIDs and gastric cancer risk. Some of these studies found a negative association (Gridley et al, 1993; Thun et al, 1993), while others did not (Isomaki et al, 1978; Schreinemachers and Everson, 1994). Results from an American population-based case–control study showed odds ratios of 0.55 for stomach adenocarcinomas and 0.88 (not statistically significant) for gastric cardia adenocarcinoma among regular users of aspirin, compared to never-users (Farrow et al, 1998). With the same exposure categories, a hospital-based case–control study from Moscow reported odds ratios of 0.49 and 1.14 (not statistically significant) for non-cardia and cardia cancer, respectively (Zaridze et al, 1999). Our findings largely confirm the results of these 2 case–control studies, although they differ with respect to the association between aspirin and cardia cancer. Despite different epidemiologic patterns for cardia and non-cardia cancer (Morales, 1997), we find it plausible that a protective effect of aspirin, mediated through its anti-inflammatory properties, would pertain to both tumour sites.

Some weaknesses of our study should be noticed. The low participation rate among our cases might have introduced bias, but would explain our results only if non-participation was strongly linked to high aspirin consumption. Since non-participation among cases was typically due to late notification to the study secretariat by the clinicians, it is unlikely that non-participating cases differed importantly from participating ones with respect to drug consumption habits. An important reason for the low participation rate in comparison with other studies is our thorough case ascertainment, which inflated the denominator. The analyses excluding subjects for whom drug-specific dosage information was not available showed somewhat stronger associations than the analyses including these individuals. Because of the questionnaire design, these individuals were those who reported use of more than one type of pain reliever. The observed difference may be due to a greater non-differential misclassification among multiple drug users, but the possibility of counteracting effects of other types of pain relievers or chance fluctuations cannot be excluded.

© 2001 Cancer Research Campaign 

An undiagnosed gastric cancer with early symptoms may have altered the use of pain relievers and thus contributed to our finding. To reduce the risk of such reverse causation, our questions regarding use of pain relievers specifically inquired about use earlier than 2 years before the interview. Furthermore, we asked about ulcer history and use of $H_2$-receptor antagonists as surrogate variables for upper gastrointestinal symptoms that may have caused a discontinued intake of pain relievers. We did not, however, find differences in aspirin use among those with symptoms as compared to those without. Recall bias is unlikely since the public is unaware of the study hypothesis, and since it is improbable that such bias would vary across histological strata. Non-differential misclassification of exposure may have been considerable but should, however, only underestimate the observed association (Rothman, 1986).

Consistent with earlier published data we observed, although with limited precision, that the inverse association between gastric cancer and aspirin appeared somewhat stronger for individuals infected with *H. pylori* (Zaridze et al, 1999). Effect modification by *H. pylori* suggests that one of the mechanisms by which aspirin confers protection may be directly or indirectly linked to *H. pylori*-associated carcinogenesis or related to factors conducive to *H. pylori* colonization. The protective effect of aspirin may be neutralizing the up-regulated COX-2 activity that has been associated with *H. pylori* infection (Sawaoka et al, 1998a).

In summary, the results of this population-based case–control study provides further support for the hypothesis that use of aspirin reduces the risk of gastric cancer, but the putative protection appeared to be less dramatic than in some recent reports.

### ACKNOWLEDGEMENTS

This work was supported by a grant from the National Cancer Institute (RO1 CA 50959).

### REFERENCES

Ekstrom AM, Eriksson M, Hansson LE, Lindgren A, Signorello LB, Nyren O and Hardell L (1999a) Occupational exposures and risk of gastric cancer in a population-based case-control study. *Cancer Res* **59**: 5932–5937

Ekstrom AM, Signorello LB, Hansson LE, Bergstrom R, Lindgren A and Nyren O (1999b) Evaluating gastric cancer misclassification: a potential explanation for the rise in cardia cancer incidence. *J Natl Cancer Inst* **91**: 786–790

Ekstrom AM, Serafini M, Nyren O, Hansson LE, Ye W and Wolk A (2000) Dietary antioxidant intake and the risk of cardia cancer and noncardia cancer of the intestinal and diffuse types: a population-based case-control study in Sweden. *Int J Cancer* **87**: 133–140

Farrow DC, Vaughan TL, Hansten PD, Stanford JL, Risch HA, Gammon MD, Chow WH, Dubrow R, Ahsan H, Mayne ST, Schoenberg JB, West AB, Rotterdam H, Fraumeni JF, Jr. and Blot WJ (1998) Use of aspirin and other nonsteroidal anti-inflammatory drugs and risk of esophageal and gastric cancer. *Cancer Epidemiol Biomarkers Prev* **7**: 97–102

Giardiello FM, Hamilton SR, Krush AJ, Piantadosi S, Hylind LM, Celano P, Booker SV, Robinson CR and Offerhaus GJ (1993) Treatment of colonic and rectal adenomas with sulindac in familial adenomatous polyposis. *N Engl J Med* **328**: 1313–1316

Graham DY ED and Evans DJ (1994) An accurate, rapid and convenient physician-office serologic test for detection of *Helicobacter pylori* infection (abstr.). *Am J Gastroenterol* **89**: 1305 (A81)

Gridley G, McLaughlin JK, Ekbom A, Klareskog L, Adami HO, Hacker DG, Hoover R and Fraumeni JF, Jr. (1993) Incidence of cancer among patients with rheumatoid arthritis. *J Natl Cancer Inst* **85**: 307–311

Hansson LE, Baron J, Nyren O, Bergstrom R, Wolk A, Lindgren A and Adami HO (1994) Early-life risk indicators of gastric cancer. A population-based case-control study in Sweden. *Int J Cancer* **57**: 32–37

Hawkey CJ (1996) Non-steroidal anti-inflammatory drug gastropathy: causes and treatment. *Scand J Gastroenterol Suppl* **220**: 124–127

Hosmer DW and Lemeshow S. (1989). *Applied logistic regression*. Wiley series in probability and mathematical statistics. Applied probability and statistics. Wiley: New York.

Isomaki HA, Hakulinen T and Joutsenlahti U (1978) Excess risk of lymphomas, leukemia and myeloma in patients with rheumatoid arthritis. *J Chronic Dis* **31**: 691–696

Jones MK, Wang H, Peskar BM, Levin E, Itani RM, Sarfeh IJ and Tarnawski AS (1999) Inhibition of angiogenesis by nonsteroidal anti-inflammatory drugs: insight into mechanisms and implications for cancer growth and ulcer healing. *Nat Med* **5**: 1418–1423

Kune GA, Kune S and Watson LF (1988) Colorectal cancer risk, chronic illnesses, operations, and medications: case control results from the Melbourne Colorectal Cancer Study. *Cancer Res* **48**: 4399–4404

Laurén (1965) The two histological main types of gastric carcinoma: diffuse and so-called intestinal-type carcinoma. *Acta Pathol Microbiol Scand* **64**: 31–49

Lupulescu A (1996) Prostaglandins, their inhibitors and cancer. *Prostaglandins Leukot Essent Fatty Acids* **54**: 83–94

Marchildon PA, Ciota LM, Zamaniyan FZ, Peacock JS and Graham DY (1996) Evaluation of three commercial enzyme immunoassays compared with the 13C urea breath test for detection of Helicobacter pylori infection. *J Clin Microbiol* **34**: 1147–1152

Marnett LJ (1992) Aspirin and the potential role of prostaglandins in colon cancer. *Cancer Res* **52**: 5575–5589

Misumi A, Murakami A, Harada K, Baba K and Akagi M (1989) Definition of carcinoma of the gastric cardia. *Langenbecks Arch Chir* **374**: 221–226

Morales TG (1997) Adenocarcinoma of the gastric cardia. *Dig Dis* **15**: 346–356

Rosenberg L, Palmer JR, Zauber AG, Warshauer ME, Stolley PD and Shapiro S (1991) A hypothesis: nonsteroidal anti-inflammatory drugs reduce the incidence of large-bowel cancer. *J Natl Cancer Inst* **83**: 355–358

Rothman KJ (1986) *Modern epidemiology*. Little Brown: Boston

Sawaoka H, Kawano S, Tsuji S, Tsuji M, Sun W, Gunawan ES and Hori M (1998a) Helicobacter pylori infection induces cyclooxygenase-2 expression in human gastric mucosa. *Prostaglandins Leukot Essent Fatty Acids* **59**: 313–316

Sawaoka H, Kawano S, Tsuji S, Tsujii M, Gunawan ES, Takei Y, Nagano K and Hori M (1998b) Cyclooxygenase-2 inhibitors suppress the growth of gastric cancer xenografts via induction of apoptosis in nude mice. *Am J Physiol* **274**: G1061–G1067

Sawaoka H, Kawano S, Tsuji S, Tsujii M, Murata H and Hori M (1998c) Effects of NSAIDs on proliferation of gastric cancer cells in vitro: possible implication of cyclooxygenase-2 in cancer development. *J Clin Gastroenterol* **27**: S47–S52

Schreinemachers DM and Everson RB (1994) Aspirin use and lung, colon, and breast cancer incidence in a prospective study. *Epidemiology* **5**: 138–146

Shiff SJ and Rigas B (1997) Nonsteroidal anti-inflammatory drugs and colorectal cancer: evolving concepts of their chemopreventive actions. *Gastroenterology* **113**: 1992–1998

Thun MJ, Namboodiri MM, Calle EE, Flanders WD and Heath CW, Jr. (1993) Aspirin use and risk of fatal cancer. *Cancer Res* **53**: 1322–1327

Tsuji S, Kawano S, Sawaoka H, Takei Y, Kobayashi I, Nagano K, Fusamoto H and Kamada T (1996) Evidences for involvement of cyclooxygenase-2 in proliferation of two gastrointestinal cancer cell lines. *Prostaglandins Leukot Essent Fatty Acids* **55**: 179–183

Zaridze D, Borisova E, Maximovitch D and Chkhikvadze V (1999) Aspirin protects against gastric cancer: results of a case-control study from Moscow, Russia. *Int J Cancer* **82**: 473–476

 *© 2001 Cancer Research Campaign*

## References

[PDF_00356] Ekström A. M., Eriksson M., Hansson L. E., Lindgren A., Signorello L. B., Nyrén O., Hardell L. (1999). Occupational exposures and risk of gastric cancer in a population-based case-control study.. Cancer Res.

[PDF_00364] Ekström A. M., Serafini M., Nyrén O., Hansson L. E., Ye W., Wolk A. (2000). Dietary antioxidant intake and the risk of cardia cancer and noncardia cancer of the intestinal and diffuse types: a population-based case-control study in Sweden.. Int J Cancer.

[PDF_00360] Ekström A. M., Signorello L. B., Hansson L. E., Bergström R., Lindgren A., Nyrén O. (1999). Evaluating gastric cancer misclassification: a potential explanation for the rise in cardia cancer incidence.. J Natl Cancer Inst.

[PDF_00368] Farrow D. C., Vaughan T. L., Hansten P. D., Stanford J. L., Risch H. A., Gammon M. D., Chow W. H., Dubrow R., Ahsan H., Mayne S. T. (1998). Use of aspirin and other nonsteroidal anti-inflammatory drugs and risk of esophageal and gastric cancer.. Cancer Epidemiol Biomarkers Prev.

[PDF_00373] Giardiello F. M., Hamilton S. R., Krush A. J., Piantadosi S., Hylind L. M., Celano P., Booker S. V., Robinson C. R., Offerhaus G. J. (1993). Treatment of colonic and rectal adenomas with sulindac in familial adenomatous polyposis.. N Engl J Med.

[PDF_00380] Gridley G., McLaughlin J. K., Ekbom A., Klareskog L., Adami H. O., Hacker D. G., Hoover R., Fraumeni J. F. (1993). Incidence of cancer among patients with rheumatoid arthritis.. J Natl Cancer Inst.

[PDF_00383] Hansson L. E., Baron J., Nyrén O., Bergström R., Wolk A., Lindgren A., Adami H. O. (1994). Early-life risk indicators of gastric cancer. A population-based case-control study in Sweden.. Int J Cancer.

[PDF_00386] Hawkey C. J. (1996). Non-steroidal anti-inflammatory drug gastropathy: causes and treatment.. Scand J Gastroenterol Suppl.

[PDF_00391] Isomäki H. A., Hakulinen T., Joutsenlahti U. (1978). Excess risk of lymphomas, leukemia and myeloma in patients with rheumatoid arthritis.. J Chronic Dis.

[PDF_00394] Jones M. K., Wang H., Peskar B. M., Levin E., Itani R. M., Sarfeh I. J., Tarnawski A. S. (1999). Inhibition of angiogenesis by nonsteroidal anti-inflammatory drugs: insight into mechanisms and implications for cancer growth and ulcer healing.. Nat Med.

[PDF_00398] Kune G. A., Kune S., Watson L. F. (1988). Colorectal cancer risk, chronic illnesses, operations, and medications: case control results from the Melbourne Colorectal Cancer Study.. Cancer Res.

[PDF_00401] LAUREN P. (1965). THE TWO HISTOLOGICAL MAIN TYPES OF GASTRIC CARCINOMA: DIFFUSE AND SO-CALLED INTESTINAL-TYPE CARCINOMA. AN ATTEMPT AT A HISTO-CLINICAL CLASSIFICATION.. Acta Pathol Microbiol Scand.

[PDF_00404] Lupulescu A. (1996). Prostaglandins, their inhibitors and cancer.. Prostaglandins Leukot Essent Fatty Acids.

[PDF_00406] Marchildon P. A., Ciota L. M., Zamaniyan F. Z., Peacock J. S., Graham D. Y. (1996). Evaluation of three commercial enzyme immunoassays compared with the 13C urea breath test for detection of Helicobacter pylori infection.. J Clin Microbiol.

[PDF_00410] Marnett L. J. (1992). Aspirin and the potential role of prostaglandins in colon cancer.. Cancer Res.

[PDF_00412] Misumi A., Murakami A., Harada K., Baba K., Akagi M. (1989). Definition of carcinoma of the gastric cardia.. Langenbecks Arch Chir.

[PDF_00415] Morales T. G. (1997). Adenocarcinoma of the gastric cardia.. Dig Dis.

[PDF_00416] Rosenberg L., Palmer J. R., Zauber A. G., Warshauer M. E., Stolley P. D., Shapiro S. (1991). A hypothesis: nonsteroidal anti-inflammatory drugs reduce the incidence of large-bowel cancer.. J Natl Cancer Inst.

[PDF_00420] Sawaoka H., Kawano S., Tsuji S., Tsuji M., Sun W., Gunawan E. S., Hori M. (1998). Helicobacter pylori infection induces cyclooxygenase-2 expression in human gastric mucosa.. Prostaglandins Leukot Essent Fatty Acids.

[PDF_00424] Sawaoka H., Kawano S., Tsuji S., Tsujii M., Gunawan E. S., Takei Y., Nagano K., Hori M. (1998). Cyclooxygenase-2 inhibitors suppress the growth of gastric cancer xenografts via induction of apoptosis in nude mice.. Am J Physiol.

[PDF_00428] Sawaoka H., Kawano S., Tsuji S., Tsujii M., Murata H., Hori M. (1998). Effects of NSAIDs on proliferation of gastric cancer cells in vitro: possible implication of cyclooxygenase-2 in cancer development.. J Clin Gastroenterol.

[PDF_00432] Schreinemachers D. M., Everson R. B. (1994). Aspirin use and lung, colon, and breast cancer incidence in a prospective study.. Epidemiology.

[PDF_00434] Shiff S. J., Rigas B. (1997). Nonsteroidal anti-inflammatory drugs and colorectal cancer: evolving concepts of their chemopreventive actions.. Gastroenterology.

[PDF_00437] Thun M. J., Namboodiri M. M., Calle E. E., Flanders W. D., Heath C. W. (1993). Aspirin use and risk of fatal cancer.. Cancer Res.

[PDF_00439] Tsuji S., Kawano S., Sawaoka H., Takei Y., Kobayashi I., Nagano K., Fusamoto H., Kamada T. (1996). Evidences for involvement of cyclooxygenase-2 in proliferation of two gastrointestinal cancer cell lines.. Prostaglandins Leukot Essent Fatty Acids.

[PDF_00443] Zaridze D., Borisova E., Maximovitch D., Chkhikvadze V. (1999). Aspirin protects against gastric cancer: results of a case-control study from Moscow, Russia.. Int J Cancer.

